# The *Eucalyptus grandis* Genome Project: Genome and transcriptome resources for comparative analysis of woody plant biology

**DOI:** 10.1186/1753-6561-5-S7-I20

**Published:** 2011-09-13

**Authors:** Alexander Myburg, Dario Grattapaglia, Gerald Tuskan, Jerry Jenkins, Jeremy Schmutz, Eshchar Mizrachi, Charles Hefer, Georgios Pappas, Lieven Sterck, Yves Van De Peer, Richard Hayes, Daniel Rokhsar

**Affiliations:** 1 Department of Genetics, Forestry and Agricultural Biotechnology Institute (FABI), University of Pretoria, Pretoria, 0002, South Africa; 2 Plant Genetics Laboratory, EMBRAPA Genetic Resources and Biotechnology - EPqB, 70770-910 Brazilia, Brazil; 3 Environmental Sciences Division, Oak Ridge National Laboratory, Oak Ridge, TN, 37831, USA; 4 HudsonAlpha Genome Sequencing Center, 601 Genome Way, Huntsville, AL 35806, USA; 5 Bioinformatics and Computational Biology Unit, Department of Biochemistry, University of Pretoria, Pretoria, South Africa; 6 Department of Plant Systems Biology, VIB, Ghent University, Technologiepark 927, 9052 Gent, Belgium; 7 Center for Integrative Genomics, Department of Molecular and Cell Biology, University of California, Berkeley, Berkeley, CA 94720, USA

## Background

The International Year of Forests - 2011 [http://www.un.org/en/events/iyof2011/] will be a milestone for forest tree genomics. The draft genome sequence of *Eucalyptus grandis* was released in January 2011 in the USA (Phytozome [http://www.phytozome.net]) and in Belgium (BOGAS, [http://bioinformatics.psb.ugent.be/webtools/bogas/]). The genome sequencing was funded by the US Department of Energy (DOE) and performed at the DOE Joint Genome Institute (JGI) in collaboration with members of the *Eucalyptus* Genome Network (EUCAGEN, [http://www.eucagen.org]) who contributed genetic materials, linkage maps, EST resources and bioinformatics support. The *E. grandis* genome together with that of *Populus trichocarpa*[1]and other woody plant genomes recently completed (e.g. *Vitis*, *Cacao*, *Prunus*, *Citrus and Malus*)will provide excellent opportunities for comparative studies of the unique biology of woody plants. Eucalypts are currently the most widely grown hardwood fibre crop in the world and eucalypt breeding programs will benefit greatly from the new genomic resources. The reference genome sequence of *Eucalyptus*, a foundation tree genus in Australia comprising more than 70% of the native forest estate, will also offer important benefits for ecological and evolutionary biology studies. We report the sequencing, assembly and annotation of the *E. grandis* genome.

## Genome sequencing and assembly

Whole-genome (8X) shotgun sequencing was performed for a partially inbred (S1), 17-year-old tree of *E. grandis* (est. genome size 640 Mbp, *n* = 11), BRASUZ1 (Suzano, Brazil). A total of 7.7 million Sanger reads (5.4 Gbp) were produced from plasmid, fosmid and BAC libraries. An inbred genotype was selected to circumvent perceived problems with the assembly of a highly heterozygous eucalypt genome. However, microsatellite genotyping showed that BRASUZ1 was much less homozygous than expected, with large parts of the genome remaining heterozygous presumably due to viability selection. This finding was confirmed during the assembly of the S1 genome - approximately 25% of the assembly occurred in two haplotypes of 3-4X coverage, while the remainder of the genome assembled into a single haplotype of 6-7X coverage. Linkage maps with over 2400 DArT and microsatellite markers were subsequently used as a framework for the assembly of 11 large chromosome scaffolds. The chromosome scaffolds contained 88% (605 Mbp) of the draft assembly, with the remainder of the assembly sequence (85 Mbp) in 4941 smaller scaffolds. Based on similarity searches with 1.6 million ESTs from BRASUZ1, it was estimated that 96% of expressed gene loci were included in the 11 chromosome assemblies.

## Genome annotation

Genome annotation was performed in parallel at the JGI and at the University of Ghent. Both annotation teams used *ab initio* and homology-based annotation approaches supported by over 4 million 454-FLX-Titanium ESTs produced by the JGI, as well as Sanger, 454 and Illumina EST data provided by collaborators. The two annotations revealed that the 11 chromosome scaffolds contain more than 90% of the predicted protein-coding loci (total 44,974 - JGI, 47,974 - UGent). More than 70% of the predicted genes had EST support and 9,961 (18%) alternatively spliced transcripts were detected. The two annotations are being compared and a joint annotation may be released for the main *E. grandis* genome paper.

## Genome duplication

The *Vitis* genome [2], representing an early diverging Rosid lineage (Vitales), was found to contain the ancient hexaploidization event shared by Rosids and Asterids, but none of the more recent genome duplications found in the Rosid lineages represented by *Arabidopsis* and *Populus*. A preliminary analysis performed at UGent of genome duplication in *E. grandis* (representing the Rosid order Myrtales) suggested that the *Eucalyptus* genome most likely contains one more recent duplication event, in addition to the paleohexaploidy event.

## Genome resequencing

*E. globulus* is a temperate eucalypt with superior wood properties compared to *E. grandis* and is viewed as the premier eucalypt species for pulping. The two species occur in different sections (*Maidenaria* and* Latoangulatae*) of the subgenus Symphyomyrtus and their genome sizes differ substantially (*E. globulus -* 530 Mbp, *E. grandis* - 640 Mbp, [3]). The JGI has performed genome-wide resequencing (>30X Illumina PE) of an *E. globulus* clone (X46, Forestal Mininco, Chile). Approximately 75% of the *E. globulus* Illumina reads mapped to the *E. grandis* reference genome and sequence analysis in these regions revealed an average sequence divergence of 1.5% between the two genomes. Other eucalypt genomes currently being resequenced by collaborators will generate a valuable resource for studies of eucalypt genome evolution.

## Transcriptome resources

The large amount of transcriptome sequence data was produced the project includes 1.9 million xylem and leaf ESTs (454 reads) from BRASUZ1 and 2.1 million 454 reads from *E. globulus* (X46) xylem and leaf tissues. Together with other large 454 datasets (e.g. [4]) and Illumina mRNA-Seq data [5]produced by collaborators, the *Eucalyptus* research community now have access to excellent transcriptome resources some of which are already available in integrated genome and transcriptome browsers (Eucspresso [http://eucspresso.bi.up.ac.za/]).

## Conclusions

The *E. grandis* genome sequence will be the first reference for the Rosid order Myrtales and will be informative for comparative genomic studies within the Eudicots. It will also deliver powerful tools for the application of genomics in eucalypt breeding programs.
